# Detection of Zika Virus Infection in Thailand, 2012–2014

**DOI:** 10.4269/ajtmh.15-0022

**Published:** 2015-08-05

**Authors:** Rome Buathong, Laura Hermann, Butsaya Thaisomboonsuk, Wiriya Rutvisuttinunt, Chonticha Klungthong, Piyawan Chinnawirotpisan, Wudtichai Manasatienkij, Ananda Nisalak, Stefan Fernandez, In-Kyu Yoon, Passakorn Akrasewi, Tanarak Plipat

**Affiliations:** Department of Disease Control, Bureau of Epidemiology, Ministry of Public Health, Nonthaburi, Thailand; Department of Virology, Armed Forces Research Institute of Medical Sciences, Bangkok, Thailand; Department of Medicine, University of Toronto, Toronto, Ontario, Canada

## Abstract

Zika virus (ZIKV) is an emerging mosquito-borne pathogen with reported cases in Africa, Asia, and large outbreaks in the Pacific. No autochthonous ZIKV infections have been confirmed in Thailand. However, there have been several cases reported in travelers returning from Thailand. Here we report seven cases of acute ZIKV infection in Thai residents across the country confirmed by molecular or serological testing including sequence data. These endemic cases, combined with previous reports in travelers, provide evidence that ZIKV is widespread throughout Thailand.

Zika virus (ZIKV) is an emerging mosquito-borne pathogen first described in 1952^1^ after being isolated from a sentinel rhesus macaque monkey in 1947 and a pool of *Aedes africanus* mosquitoes in 1948 from the Zika forest in Uganda. Since it was first reported, only a small number of cases had been described in Africa and Asia until 2007 when there was a large outbreak on Yap Island in the Federated States of Micronesia.^2^,^3^ In October 2013, ZIKV was detected in French Polynesia where at least 396 laboratory-confirmed cases have occurred and an estimated 29,000 people (∼10% of the total population) have sought medical care for suspected Zika illness.^4^ The affected areas in the Pacific have expanded to include the Cook Islands, New Caledonia, and Easter Island.^5^

ZIKV is an approximately 11-kb single-stranded, positive-sense ribonucleic acid (RNA) virus from the Flaviviridae family, most closely related to the Spondweni virus. Two major lineages, African and Asian, have been identified through phylogenetic analyses.^2^,^6^,^7^ Transmission likely occurs via mosquito vectors from the *Aedes* genus of the Culicidae family in a sylvatic cycle involving nonhuman primates,^8^ although antibodies have been detected in a number of other mammals (i.e., water buffalo, elephants, zebras).^4^ Humans have been suggested as primary amplification hosts in areas where there are no nonhuman primates.^3^

A substantial proportion of ZIKV infections are subclinical,^3^,^9^ but patients may also present with clinical symptoms similar to other arboviral infections (e.g., dengue and chikungunya). Commonly reported symptoms in the Yap Island and French Polynesian outbreaks included rash (90–95%), fever (65–73%), arthralgia (65–70%), and non-purulent conjunctivitis (55–63%).^3^,^9^ Symptoms tend to be mild with no reported deaths. However, there is a concern that ZIKV infection may be related to an increase in cases of Guillain–Barre syndrome seen during the French Polynesian outbreak.^10^

Diagnosis of ZIKV infection is hampered by serological cross-reactivity with other flaviviruses, and relies on the detection of ZIKV RNA in blood through ZIKV-specific reverse transcription polymerase chain reaction (RT-PCR) or pan-flavivirus PCR amplification followed by sequencing or viral isolation. The U.S. Centers for Disease Control and Prevention (CDC) proposed a case classification scheme during the epidemic in Yap in 2007, which combines molecular and serological methods.^3^

In Thailand, no autochthonous ZIKV infections have been confirmed in Thai residents. However, several Zika cases have been reported in travelers returning from Thailand. In early 2013, ZIKV RNA was detected in the blood of a 45-year-old Canadian woman who had recently returned from a vacation in southern Thailand.^11^ Another case of ZIKV infection was reported in an adult male from Germany who had developed symptoms 12 days after arrival in Thailand while visiting a number of islands in southern Thailand.^12^ Most recently, a suspected case of ZIKV infection was reported in a 41-year-old Japanese male traveler to southern Thailand who had serological evidence of ZIKV infection in an acute blood sample.^13^ Acute ZIKV infections in southeast Asia have also been reported from Indonesia^14^ and Cambodia.^15^ Indirect serological evidence of ZIKV infection has been documented in the past in non-acute blood samples from Thailand, Vietnam, Malaysia, Indonesia, and the Philippines.^8^

The Thailand Ministry of Public Health (MOPH) collects and tests samples during outbreak investigations for public health. Full ethical review was not required; however, the purpose of the investigation was provided and verbal consent from the patient was obtained. The MOPH routinely tests samples from patients presenting with a maculopapular rash and/or fever for immunoglobulin M (IgM) antibodies by enzyme-linked immunosorbent assay (ELISA) to measles, rubella, chikungunya, and dengue viruses in convalescent serum samples. Acute samples (collected within 7 days of symptom onset) are also tested by RT-PCR for detection of dengue virus (DENV)^16^ and chikungunya virus (CHIKV)^17^ RNA.

In May 2013, the Thai MOPH was notified that ZIKV infection had been confirmed in a Canadian traveler to Thailand^11^ prompting the MOPH to further evaluate a number of acute illness samples from outbreak investigations for evidence of ZIKV infection. A review of outbreak investigations conducted by the Thai MOPH throughout Thailand from January 2012 to December 2014 was undertaken. An outbreak was considered for further evaluation when individuals in the cluster had two of the following symptoms: 1) rash, 2) conjunctivitis, or 3) arthralgia, and the acute serum sample was negative for DENV and CHIKV and the IgM antibodies to rubella and measles were not detected in the convalescent sera. Four clusters out of 175 were identified for further evaluation for ZIKV infection.

Here, we report seven cases of acute ZIKV infection in Thai residents from different regions of the country confirmed by molecular or serological testing including one viral isolate with sequence data. These endemic cases, combined with previous ZIKV infection reported in travelers, provide evidence that ZIKV is widespread throughout Thailand.

Table 1 provides the characteristics of the samples and the results of the diagnostic assays. A total of 61 samples representing 38 subjects from the four outbreak clusters were available for further testing. Of the samples obtained before 2014, 54 were sent to the U.S. CDC in Fort Collins, CO, for in-house ZIKV serologic testing including IgM ELISA and plaque reduction neutralization test (PRNT)^3^ while seven samples (obtained in 2014) were tested by the Thai CDC once ZIKV diagnostic PCR testing was available. PRNT testing was not completed on these samples. Acute samples were tested by real-time RT-PCR targeting the premembrane (prM)/envelope gene at the Armed Forces Research Institute of Medical Sciences (AFRIMS).^2^

Three samples taken from Ratchaburi province, Thailand, were positive for ZIKV infection based on serologic studies whereas ZIKV RNA was detected in the other four samples. The 77 base pair RT-PCR amplicon, used for ZIKV diagnosis from a sample from Sisaket province, was sequenced and found to have high nucleotide identity (between 90% and 100%) to the prM/E region of ZIKV sequences reported in GenBank. Subsequently, three serum samples collected in Phetchabun province that were positive or equivocal for ZIKV by PCR were sent for virus isolation by intrathoracic inoculation of *Toxorhynchites splendens* mosquitoes followed by inoculation of mosquito-derived C6/36 cells. Typing ELISA using the universal flavivirus (4G2 monoclonal) antibody and DENV serotypes 1, 2, 3, and 4 specific monoclonal antibodies was also completed on the supernatants from the C6/36 cells.^18^,^19^ One supernatant sample positive for 4G2 antibody, but negative for DENV antibodies, underwent next-generation sequencing (Illumina MiSeq Platform, Illumina, Hayward, CA). De novo assembly produced a 10,685 nucleotide (nt) consensus sequence (depth of coverage greater than 10,000). Maximum likelihood (ML) phylogenetic analysis of a 789-bp partial sequence of ZIKV nonstructural protein 5 (ZIKV NS5) (GenBank accession no. KM851039) indicated that the isolate belonged to the ZIKV Asian lineage, which includes strains from the Canadian traveler to Thailand (2013)^11^ and strains from Cambodia (2010),^15^ Micronesia (2007),^2^ and French Polynesia (2013)^20^ (Figure 1). Pairwise genetic distance calculation illustrated that the isolate was most closely related to the French Polynesia 2013 strain (*p*-distance = 0.009) with approximately 99% nucleotide identity (782/789).

**Figure 1.**
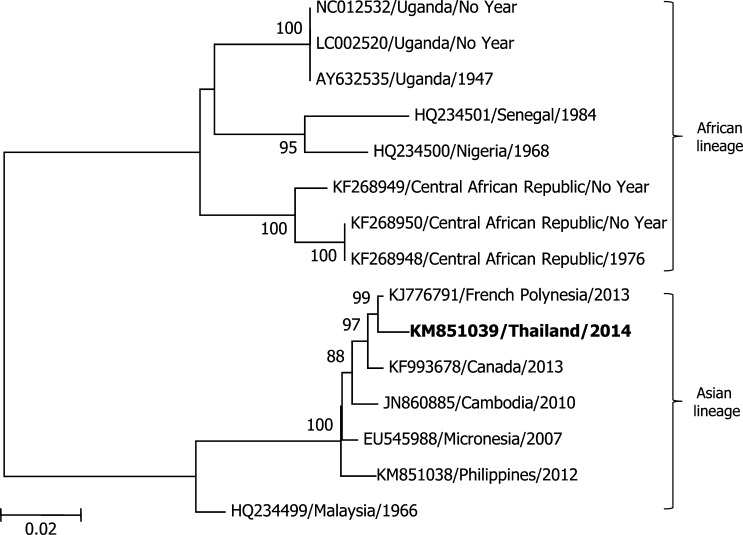
Maximum likelihood phylogenetic trees of fragments of Zika virus nonstructural protein 5 (ZIKV NS5) utilizing GTR+G+I model with 14 reference ZIKV strains from GenBank. The contig sequences, obtained from de novo assembly and BLASTn, of KM851039/Thailand/2014 were analyzed against eight references from the African lineage (Accession numbers: KF268948, KF268950, KF268949, LC002520, AY632535, NC012532, HQ234500, and HQ234501) and six references from the Asian lineage (Accession numbers: KJ776791, JN860885, EU545988, HQ234499, KF993678, and KM851038). The scale of genetic distance equal to 0.02 is indicated in the bottom left of the panel. Bootstrap ≥ 70 is demonstrated next to the node of the tree. The year of collection is unknown for a number of African strains. Drawing is not to scale.

Full clinical information was available for five cases. There were four female patients and one male patient with an average age of 25 years (range: 12–39 years). Clinical presentation was mild and nonspecific. All subjects presented with fever and maculopapular rash. Other symptoms included sore throat (2), arthralgia (2), myalgia (1), rhinorhea (1), and headache (1). Of note, only two patients complained of conjunctivitis, which was less than previously reported in ZIKV cases outside Africa^3^,^4^,^9^ including the travelers to Thailand with ZIKV infection.^11^,^12^

Despite the isolate from Phetchabun (2014) having a closer relationship to the French Polynesian strain (2013) than the strain from the Canadian traveler (2013) or the strain from Cambodia (2010), these three ZIKV isolates are all part of the ZIKV Asian lineage with Micronesia (2007) and Philippines (2012). The phylogenetic analysis suggests that strains collected in Asia and the Pacific are all closely related. It is possible that these strains may have been co-circulating in this region for several years, but remained undetected as symptoms may have been attributed to other known endemic arboviruses with similar presentations, such as DENV and CHIKV. Alternatively, ZIKV may have been spread to each location successively as samples were collected at different time points.

Our report identifies a number of cases of confirmed ZIKV infection from different provinces across Thailand in patients with no recently reported travel outside their home province. When combined with previous reports of ZIKV infection in travelers to Thailand, it was clear that ZIKV has a widespread distribution across the country (Figure 2). In future, increased surveillance using pan-flavivirus or ZIKV-specific molecular testing may lead to more frequent identification of ZIKV infections, which will help better describe the clinical spectrum of ZIKV infection, the distribution in Thailand, and the potential effects of co-circulation with other endemic flaviviruses.

**Figure 2.**
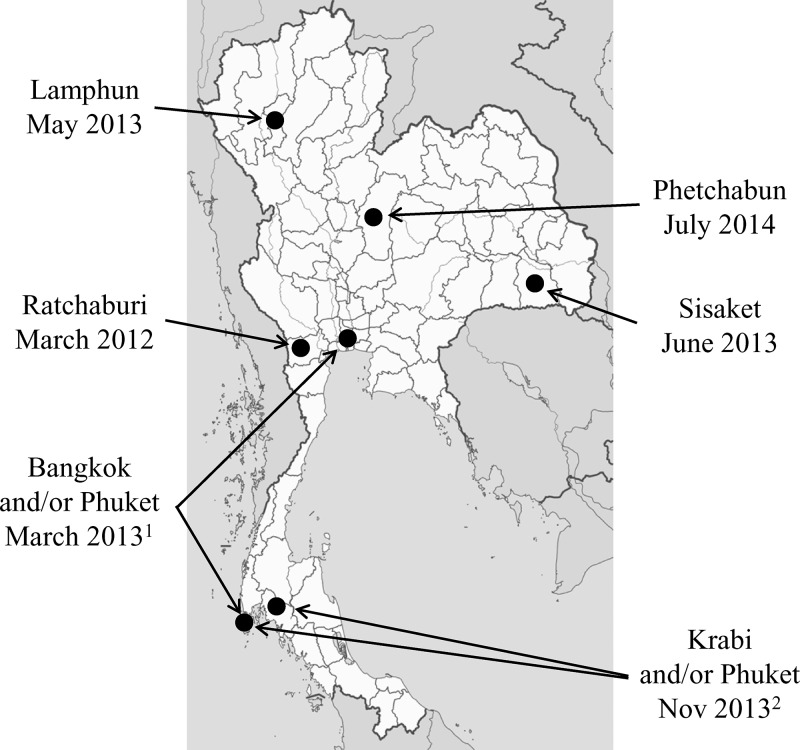
Map of Thailand showing locations of confirmed Zika virus (ZIKV) infections. 1ZIKV infection in Canadian traveler to Bangkok and Phuket provinces.^11^ ^2^ZIKV infection in German traveler to Phuket and Krabi provinces.^12^

## Figures and Tables

**Table 1:** Summary of sample testing

Date	Province	Age	Gender	DOI (days)	DENV RT-PCR	ZIKV RT-PCR	DENV IgM	DENV IgG	ZIKV IgM	DENV PRNT	ZIKV PRNT	Interpretation*
March 2012	Ratchaburi	18	M	7	ND	Negative	Positive	Negative	Positive	160	1,280	ZIKV
March 2012	Ratchaburi	12	F	9	ND	ND	Negative	Negative	Positive	20	1,024	ZIKV
March 2012	Ratchaburi	32	F	16	ND	ND	Negative	Negative	Positive	80	10,240	ZIKV
September 2013	Lamphun	9	F	1	Negative	Positive	Negative	Negative	Negative	ND	ND	ZIKV
September 2013	Sisaket	53	F	3	Negative	Positive	Negative	Negative	ND	ND	ND	ZIKV
July 2014	Phetchabun	39	F	1	Negative	Positive	ND†	ND†	ND	ND	ND	ZIKV‡
July 2014	Phetchabun	24	F	3	Negative	Positive	ND†	ND†	ND	ND	ND	ZIKV

DENV = dengue virus; DOI = date of illness; ND = not determined; PRNT = plaque reduction neutralization test; RT-PCR = reverse transcription polymerase chain reaction; ZIKV = Zika virus.

* Interpretation: a sample was considered positive if ZIKV RNA was detected (positive PCR) or immunoglobulin M (IgM) antibody was present against ZIKV, and ZIKV PRNT was ≥ 20 with ZIKV PRNT:DENV PRNT ratio ≥ 4.

† Both samples collected from Phetchabun province were also negative for dengue virus nonstructural protein 1 (DENV NS1) antigen testing.

‡ Virus for sequencing was isolated from this sample by intrathoracic inoculation of *Toxorhynchites splendens* mosquitoes from the serum followed by inoculation of C6/36 cells.
